# Meta-analysis of risk factors for CCLNM in patients with unilateral cN0 PTC

**DOI:** 10.1530/EC-20-0058

**Published:** 2020-04-08

**Authors:** Wei Sun, Boyuan Zheng, Zhihong Wang, Wenwu Dong, Yuan Qin, Hao Zhang

**Affiliations:** 1 Department of Thyroid Surgery, The First Hospital of China Medical University, Shenyang, Liaoning Province, China

**Keywords:** thyroid cancer, papillary, lymphatic metastasis, risk factors

## Abstract

**Background:**

In patients with papillary thyroid cancer (PTC) with clinical negative central lymph nodes (cN0), the use of prophylactic central lymph node dissection remains controversial. Contralateral central lymph node metastasis (CCLNM) occurs in 3.88–30.63% of patients with cN0 PTC. Therefore, the present meta-analysis aimed to obtain evidence for CCLNM risk factors in unilateral cN0 PTC.

**Materials and methods:**

Relevant studies were identified in the PubMed, SCIE, and Wanfang databases up to Oct 31, 2019. The included patients had undergone lobectomy or total thyroidectomy with bilateral central lymph node dissection and were diagnosed pathologically with PTC. Revman 5.3 software was applied for statistical analysis.

**Results:**

Thirteen studies comprising 2449 patients were included. The factors associated with increased CCLNM risk in patients with cN0 disease were: age <45 years (odds ratio (OR) = 1.89, 95% CI = 1.43–2.49, *P* < 0.00001), male sex (OR = 1.67, 95% CI = 1.24–2.24, *P* = 0.0007), extrathyroidal extension (OR = 1.63; 95% CI = 1.17–2.28; *P* = 0.004), tumor size ≥1 cm (OR = 2.63, 95% CI 1.85–3.74, *P* < 0.00001), lymphovascular invasion (OR = 4.27, 95% CI = 2.47–7.37, *P* < 0.00001), and ipsilateral central lymph node metastasis (OR = 11.42, 95% CI = 5.25–24.86, *P* < 0.00001). However, no association was found for capsular invasion, multifocality, or Hashimoto thyroiditis.

**Conclusion:**

The meta-analysis identified that age <45 years, tumor ≥1 cm, male sex, lymphovascular invasion, extrathyroidal extension, and ipsilateral central lymph node metastasis are related to CCLNM in patients with unilateral CN0 PTC. These factors should influence the use of prophylactic central lymph node dissection in this group of patients.

## Introduction

The most prevalent endocrine malignancy is thyroid cancer, which accounts for 1% of all malignancies. Recently, morbidity caused by thyroid cancer has increased by three-fold (1, 2). Histologically, the most common type of thyroid cancer is papillary thyroid carcinoma (PTC), representing >90% of all thyroid cancers (1). The prognosis of PTC is generally favorable and the overall prognosis is excellent. The 5-, 10-, and 20-year survival rates of PTC are 94, 89, and 87%, respectively (3, 4). Patients with PTC develop cervical lymph node metastases in approximately 30–80% of cases (5, 6). However, in patients with PTC, the significance of lymph node metastasis is a matter of debate. For example, some studies reported that relapse is affected by lymph node metastasis, whereas survival is not (7). However, a recent large single-institution study showed that the outcome of PTC could be predicted using the ratio of the number of positive lymph nodes to the total number of excised nodes (8).

Therapeutic central neck dissection is acceptable in patients with clinically positive PTC. However, it remains controversial as to whether patients with clinically negative central lymph nodes (cN0) should receive prophylactic central lymph node dissection (PCLND). At present, there is no convincing evidence that patient prognosis is improved using PCLND. Nixon *et al*. observed 100% 5- and 10-year disease-specific survival rates among patients with PTC who did not receive PCLND (9). In addition, dynamic observation of central lymph nodes is considered safe and should be performed for all patients with PTC before and during surgery to ensure that they are free from central neck metastasis (10). However, other studies have reported benefits of PCLND in patients with cN0. For example, PCLDN can help to accurately diagnose tumor-node-metastasis (TNM) staging, help decide on the use of thyroid-stimulating hormone (TSH) suppression therapy or radioactive iodine (RAI) therapy, and predict the possibility of lateral lymph node metastases (11, 12, 13). Other studies have reported that PCLND can reduce thyroglobulin levels during postoperative follow-up, reduce postoperative recurrence, and improve disease-specific survival (14, 15, 16).

PTC initially spreads from the thyroid gland to lymph nodes in the pretrachea, the ipsilateral tracheoesophageal groove, and to nodes in the ipsilateral neck and mediastinum (17, 18). Contralateral central compartment and contralateral neck and skip metastases (negative central and positive lateral or mediastinal lymph nodes) are generally rare. However, in PTC, the frequency of central lymph node metastasis is high, leading to 3.88–30.63% of patients with unilateral cN0 disease developing contralateral central lymph node metastasis (CCLNM) (19, 20). If bilateral PCLND was applied to all of the PTC patients, the rates of hypoparathyroidism and vocal cord palsy would inevitably increase (21, 22). To reveal central lymph node metastases, preoperative imaging such as contrast-enhanced CT and ultrasound (US) are used widely. However, both US and enhanced CT are not particularly accurate, showing sensitivities of 27.5–38% and 38.9–50%, respectively (23, 24, 25). Therefore, treatment options for contralateral central lymph node compartments should be considered carefully to balance the risks and benefits of PCLND. However, although risk factors have been identified for patients with cN0 disease, the results were inconsistent, probably because of the small sample sizes used. Therefore, in the present study, a meta-analysis was used to assess the clinical features of CCLNM in patients with cN0 PTC.

## Methods

This meta-analysis was performed according to the guidelines of the preferred reporting items for systematic reviews and meta-analyses (PRISMA) statement (26).

### Search strategy

The Wan Fang, Web of science, and PubMed databases were subjected to a comprehensive literature search for studies published up to Oct 31, 2019 using the key words ((((((((central) OR central compartment) OR level VI) OR paratracheal)) AND lymph node) AND contralateral)) AND (((((((thyroid cancer) OR thyroid carcinoma) OR thyroid neoplasm)) AND papillary)) OR PTC). The two authors (Sun W and Zheng BY) independently completed the selection process. Differences were resolved via discussion

### Selection criteria

The meta-analysis included studies that met these criteria: (a) Retrospective or prospective cohort studies; (b) the lesion was only located in one side of the thyroid lobe, and no nodule was found in the other side of the thyroid lobe; (c) only patients who underwent unilateral lobectomy or total thyroidectomy plus bilateral PCLND, and PTC was confirmed pathologically intraoperatively or postoperatively; (d) patients with PTC and clinically negative neck nodes; and (e) the medical records were complete to allow data extraction. The exclusion criteria comprised: (a) Patients with a history of other thyroid malignancies or previous treatment for head and neck cancer; (b) case reports, reviews, conference abstracts, letters to the editor, and so on; and (c) patients whose family has a history of thyroid cancer.

### Data extraction and quality assessment

Two authors (Sun W and Zheng BY) extracted the data independently. Detailed information was recorded on first author, publication years, author country, study design, PTC/PTMC, case number, CCLNM rate, and surgical intervention. Independent records were made for nine possible risk factors of CCLNM and the corresponding numbers of patients. The nine risk factors comprised sex, age, tumor size, multifocality, extrathyroidal extension, capsular invasion, lymphovascular invasion, Hashimoto thyroiditis, and ipsilateral central lymph node metastasis. We included data on the number of CCLNMs. To assess the quality of the included studies, we used the Newcastle-Ottawa quality assessment scale.

### Statistical analysis

Review Manager 5.3 (https://community.cochrane.org) was used to perform all the statistical analyses. The results are presented as the odds ratios (ORs) with 95% CIs. Unless otherwise specified, *P* values <0.05 are considered statistically significant. In addition, the *I*^2^ statistics and the Q test were used to quantify heterogeneity. When *I*^2^ was less than 50% and *P* was greater than 0.1, a fixed effect model was used. In other cases, we used a random effect model. To test for possible publication bias, Begg’s funnel plots were used.

## Results

**Figure 1:**
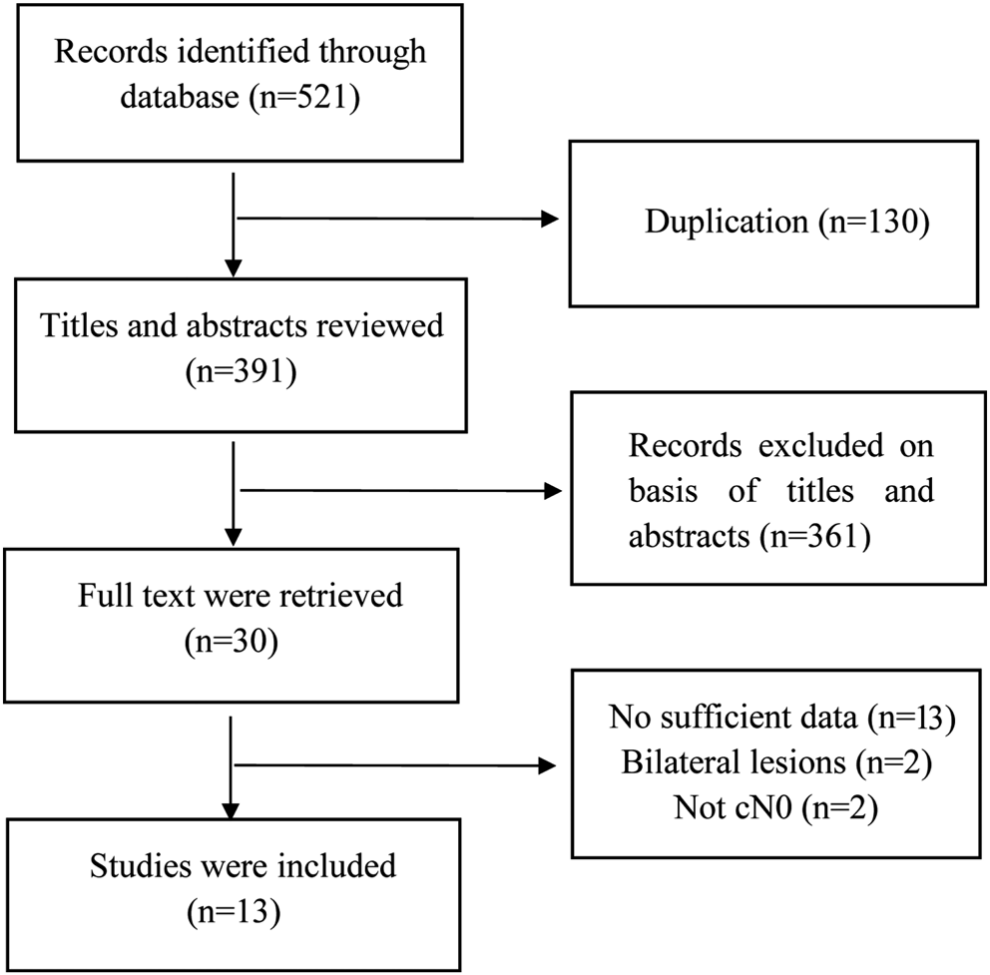
Flow chart of the study selection process.

Database screening identified 521 studies, of which 130 were excluded because of repetition and language. Next, 361 studies were excluded after careful scanning of their titles and abstracts because they comprised case reports, reviews, and unrelated studies. Thirty articles remained for full text evaluation. After applying the inclusion criteria, the meta-analysis included 13 studies comprising 2449 patients, among which nine studies were retrospective and four studies were prospective. The basic characteristics of the articles are shown in Table 1. The Begg’s funnel chart is provided in the Supplementary information (see section on supplementary materials given at the end of this article). Figure 1 shows a flowchart of the selection process.

**Table 1:** Basic characteristics of the included studies.

Author	Year	Country	Study design	PTC/PTMC	Case number	Contralateral metastasis	Operation	Quality assessment
Ahn BH (29)	2014	Korea	prospective	PTC	368	7.10%	TT + bilateral CLND	8
Chen Q (46)	2015	China	retrospective	PTC	218	13.30%	TT + bilateral CLND	7
Eun YG (47)	2014	Korea	retrospective	PTC	140	10.00%	TT + bilateral CLND	8
Gu Z (50)	2016	China	retrospective	PTC	46	21.74%	TT/lobectomy + bilateral CLND	6
He W (51)	2017	China	retrospective	PTC	149	23.49%	TT + bilateral CLND	7
Ji YB (19)	2016	Korea	retrospective	PTC	361	3.88%	TT + bilateral CLND	7
Koo BS (20)	2009	Korea	prospective	PTC	111	30.63%	TT + bilateral CLND	8
Lim YC (48)	2009	Korea	retrospective	PTMC	86	10.47%	TT + bilateral CLND	7
Miao SS (49)	2014	China	prospective	PTC	184	16.30%	TT + bilateral CLND	6
Qin A (52)	2017	China	retrospective	PTC/PTMC	245	15.10%	TT + bilateral CLND	7
Roh JL (6)	2015	Korea	prospective	PTC	184	9.78%	TT + bilateral CLND	8
Yoo HS (53)	2017	Korea	retrospective	PTC	215	4.19%	TT + bilateral CLND	7
Zhang L (54)	2016	China	retrospective	PTC	169	14.20%	TT + bilateral CLND	7

### Age

**Figure 2:**
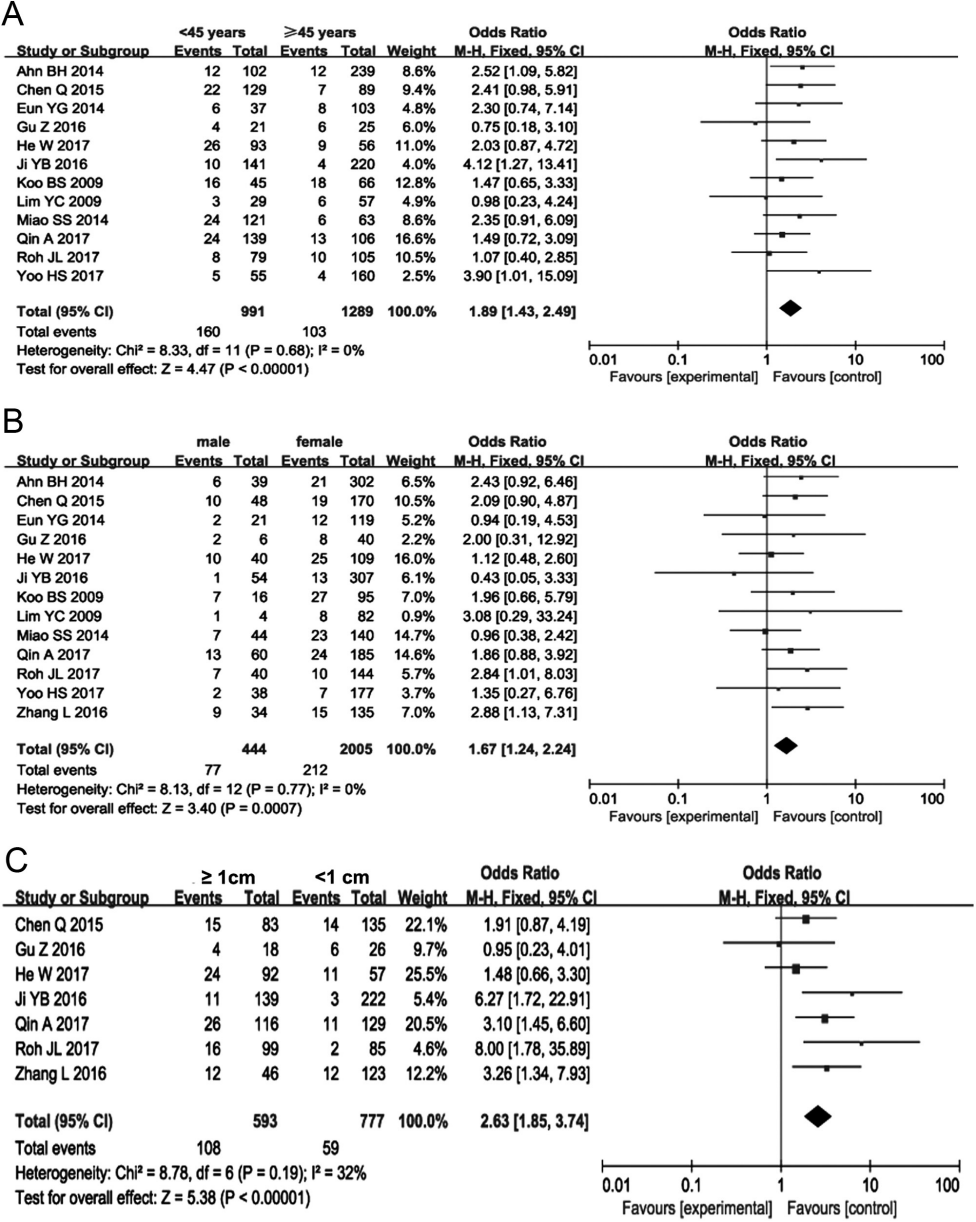
Forest plots of the association between age, sex, size, and CCLNM in cN0 PTC. (A) Age; (B) sex; (C) size.

Heterogeneity was assessed using a fixed-effects model (*P* = 0.68, *I*^2^ = 0%). To analyze the association with age and CCLNM, 45 years old was used as the cut off. Among patients with cN0 PTC, 16.15% of patients who were less than 45 years old and 7.99% of patients who were ≥45 years old had CCLNM. Upon meta-analysis, these data showed that an increased rate of CCLNM was associated with age <45 years old in patients with cN0 PTC (OR = 1.89, 95% CI = 1.43–2.49, *P* < 0.00001) (Fig. 2A) (Supplementary Fig. 1).

### Sex

Heterogeneity was assessed using a fixed-effects model (*P* = 0.77, *I* ^2^ = 0%). Among patients with cN0 PTC, 17.34% of men and 10.57% of women had CCLNM. Thus, male patients with cN0 PTC had a significantly higher incidence of CCLNM than female patients (OR = 1.67, 95% CI = 1.24–2.24, *P* < 0.00001) (Fig. 2B) (Supplementary Fig. 2).

### Tumor size

Seven studies were included in the analysis of the influence of tumor size in patients with PTC. A fixed-effects model was used in this analysis (*P* = 0.19, *I*^2^ = 32%). The incidence of CCLNM was 18.21% in patients with a tumor size ≥1 cm and 7.59% in patients with a tumor size <1 cm. Thus, CCLNM was associated significantly with tumor size ≥1 cm in patients with cN0 PTC (OR = 2.63, 95% CI = 1.85–3.74, *P* < 0.00001) (Fig. 2C) (Supplementary Fig. 3).

### Multifocality

**Figure 3:**
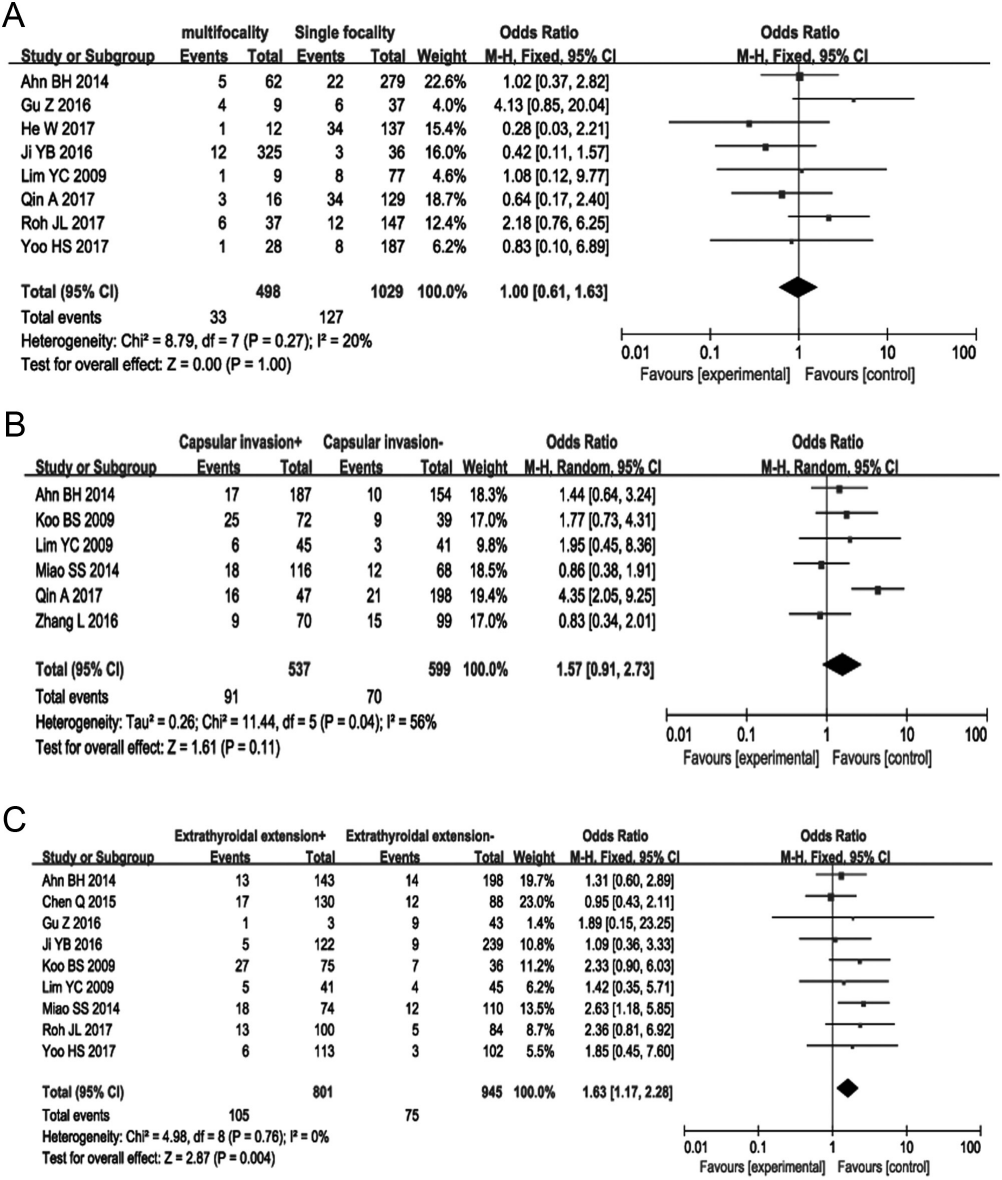
Forest plots of the association between multifocality, capsular invasion extrathyroidal extension and CCLNM in cN0 PTC. (A) Multifocality; (B) capsular invasion; (C) extrathyroidal extension.

Nine articles, including 1926 patients, were included in the relationship between CCLNM and multifocality in the unilateral lobe. Meta-analysis with a fixed effect model was used (*P* = 0.27, *I* ^2^ = 20%). The results showed that there was no statistically significant difference in the rate of CCLNM between the patients with multifocality and single focality (OR = 1.00, 95% CI = 0.61–1.63; *P* = 1.00) (Fig. 3A) (Supplementary Fig. 4).

### Capsular invasion

The data from six included studies was assessed using a random-effects model (*P* = 0.04, *I*^2^ = 56%). The results showed that CCLNM was not associated with capsular invasion in patients with cN0 PTC (OR = 1.57, 95% CI = 0.91–2.73, *P* = 0.11) (Fig. 3B) (Supplementary Fig. 5).

### Extrathyroidal extension

To analyze the association of extrathyroidal extension and CCLNM, 9 articles containing 1746 patients were included. This analysis used a fixed-effects model (*P* = 0.76, *I*^2^ = 0%). The results showed that the proportion of CCLNM in patients with extrathyroidal extension was higher than that in patients without extrathyroidal extension (OR = 1.63; 95% CI = 1.17–2.28; *P* = 0.004) (Fig. 3C) (Supplementary Fig. 6).

### Lymphovascular invasion

**Figure 4:**
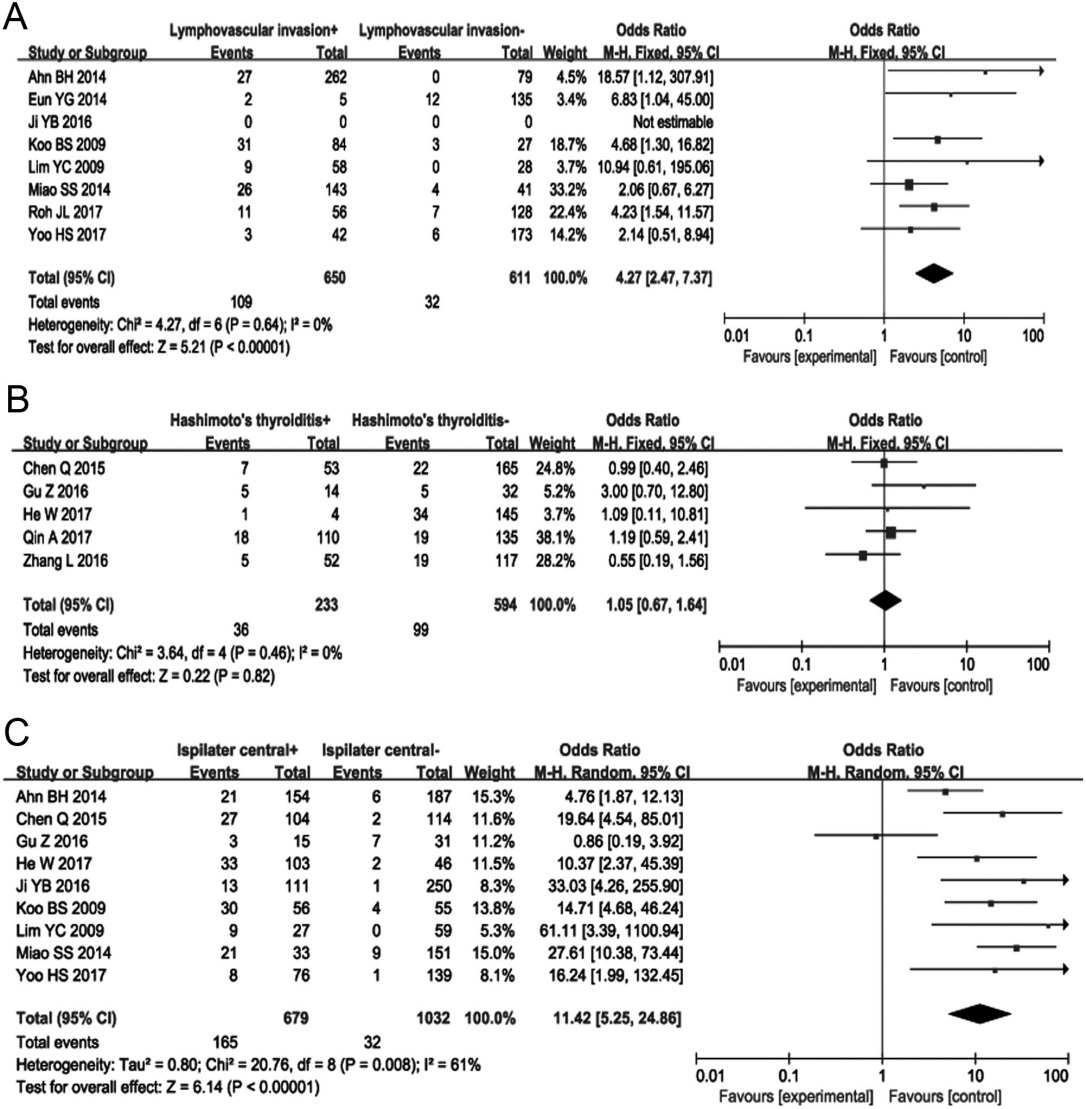
Forest plots of the association between lymphovascular invasion, Hashimoto thyroiditis, ipsilateral central lymph node metastasis and CCLNM in cN0 PTC. (A) Lymphovascular invasion; (B) Hashimoto thyroiditis; (C) ipsilateral central lymph node metastasis.

Lymphovascular invasion was analyzed in eight articles using a fixed effects model (*P* = 0.64, *I* ^2^ = 0.0%). Lymphovascular invasion was associated with a 3.4-fold higher risk of CCLNM in patients with cN0 PTC (OR = 4.27, 95% CI = 2.47–7.37, *P* < 0.00001) (Fig. 4A) (Supplementary Fig. 7).

### Hashimoto thyroiditis

Five studies were included in the analysis of data involving Hashimoto’s thyroiditis, which used a fixed-effects model (*I* ^2^ = 0%, *P* = 0.46). Hashimoto thyroiditis was not associated with co-existing CCLNM (OR = 1.05, 95% CI = 0.67–1.64, *P* = 0.82) (Fig. 4B) (Supplementary Fig. 8).

### Ipsilateral central lymph node metastasis

Nine articles and 1711 patients were included to analyze the relationship of ipsilateral lymph node metastasis and CCLNM. Heterogeneity was assessed using a random-effects model (*P* = 0.008, *I*^2^ = 61%). The results showed that the proportion of CCLNM was 23.30% in patients with ipsilateral CLNM, which was significantly higher than that in patients without ipsilateral lymph node metastasis (OR = 11.42, 95% CI = 5.25–24.86, *P* < 0.00001) (Fig. 4C) (Supplementary Fig. 9).

## Discussion

Most cases of PTC are not aggressive and have an optimistic prognosis; however, PTC shows a tendency for early spread to the central lymph node. The indications and prognostic effects of PCLND remain controversial. For differentiated thyroid cancer (DTC), the American Thyroid Association (ATA) guidelines recommend PCLND only for patients with stage T3–4 disease with cN0 and all cN1b disease; however, a considerable number of surgeons, especially in China and Japan, still believe that it is valuable to perform ipsilateral central lymph node dissection while excising thyroid lesions (27). PCLND can help to make postoperative TNM staging and recurrence risk stratification more accurate, and aid the identification of patients that are suitable for RAI treatment (11, 12, 13). For experienced surgeons, ipsilateral central lymph node dissection does not increase the incidence of surgical complications and operation time (14). CCLNM is not common compared with ipsilateral central lymph node metastasis; however, studies have reported that CCLNM occurs in 3.88–30.63% in patients with cN0 PTC (19, 20). The omission of lymph nodes is bound to increase the rate of reoperation and affect patient prognosis (28). However, it is worth noting that there is still no effective method to predict which patients have lymph node metastasis in the contralateral central lymph node. The sensitivities of US and contrast enhanced CT are low. In addition, fine-needle aspiration cytology is associated with false negative rates of 6–8%, which can be up to 20% in non-diagnostic specimens (29). Thus, it is important to use clinical pathological data to predict which patients could have CCLNM. The outcome of this study is based on soft data (e.g., the presence or absence of metastases in the contralateral central lymph nodes), but not on the recurrence rate or survival.

Age is an important prognostic factor for DTC, and the prognosis of patients with DTC worsens with increasing age (30). However, many studies have reported that young age is a risk factor for lymph node metastasis (31, 32). In the present meta-analysis, age less than 45 years was identified as an important risk factor for CCLNM. Therefore, contralateral central lymph node dissection should be performed in patients younger than 45 years old because of their probability of good prognosis.

Thyroid cancer is a female-dominant sex-dimorphic cancer and the incidence of PTC among woman is nearly three-times higher than that in men (33). However, male patients are also more likely to have unhealthy lifestyles and harmful environmental factors, for example, drinking alcohol and smoking (34). Men’s poorer prognosis compared with that of women has been reported by several studies (35, 36). The meta-analysis indicated that in patients with cN0 PTC, male sex is a significant risk factor for CCLNM.

The National Thyroid Cancer Treatment Cooperative Study (NTCTCS), the American Joint Committee on Cancer (AJCC), and MACIS (metastasis, age, completeness of resection, invasion, size) frequently use tumor size in their staging systems (37). Risk stratification has used 1 cm as most common cut-off and is accepted as a risk factor for CLNM (6, 38). Larger tumors are more likely to be aggressive, undergo lymph node metastasis, and result in poor prognosis. In the present meta-analysis, patients with cN0 PTC with a tumor size ≥1 cm had a 2.4-fold increased risk of CCLNM.

It is a matter of debate as to whether multifocality is associated with CCLNM in patients with cN0 PTC. Multifocal PTC is believed to be more aggressive compared with unifocal PTC and is an independent risk factor for PTC recurrence after total thyroidectomy (39). However, a propensity score-matching analysis indicated that multifocality is not an independent prognostic factor in PTC (40). Similarly, the results of the present study indicated that multifocality is not a risk factor for CCLNM.

Whether patients with Hashimoto thyroiditis (HT) are predisposed to develop thyroid nodules and thyroid cancer is unclear. The coexistence of HT has been suggested as not associated with CCLNM in patients with PTC or PTMC (41). A previous meta-analysis revealed that patients with PTC and coexisting HT exhibited less aggressive clinicopathological characteristics, such as lower rates of lymph node metastasis and extrathyroidal extension, and experienced longer recurrence-free survival compared with patients with PTC without HT (42). The results of the present meta-analysis suggested no association between HT and CCLNM in patients with cN0 PTC. The discrepancies between the present findings and those of previous studies might reflect different selection criteria and study designs.

The course of PTC is slow and long. The tumor may break through the glandular capsular and invade the capsular or surrounding muscles and blood vessels, and the recurrent laryngeal nerve. Extrathyroid extension and lymphovascular invasion are believed to have an marked effect on lymph node metastasis and poor prognosis (43). However, whether capsular invasion is a prognostic factor in PTC remains controversial. Studies reported that capsular invasion does not seem to cause death but is an independent risk factor for regional recurrence (44, 45). The results of the present meta-analysis showed that CCLNM was more likely to occur in patients with extrathyroid extension and lymphovascular invasion. However, capsular invasion was not a risk factor associated with CCLNM.

In the present study, although occult CCLNM was rare, it was associated with the occurrence of ipsilateral central compartment metastasis. The presence of ipsilateral central compartment metastasis exhibited a 7.83-fold increased risk of CCLNM compared with patients without this type of metastasis. Thus, for this subset of patients, ipsilateral PCLND could represent an appropriate prophylactic procedure. If frozen biopsy shows metastasis to the ipsilateral central lymph node, CCLNM dissection can be performed.

The present study has some limitations. First, the studies that we included were not randomized case-control trials. Second, despite lymph node metastasis being closely associated with the tumor location in the thyroid gland, no tumor was analyzed for its location in this meta-analysis. Third, the majority of the patients analyzed from the included studies were from Asia. Fourth, prospective and retrospective studies were mixed in this study. Five, we were limited to the original data from the included studies; therefore, we could not obtain enough data to perform multivariate analysis.

In summary, the meta-analysis identified age, gender, tumor size, extrathyroid expansion, and lymphatic invasion as important risk factors for CCLNM in patients with cN0 PTC. In patients with cN0 PTC, CCLNM did nor correlate with multifocality, capsule invasion, or Hashimoto thyroiditis.

## Supplementary Material

Supporting Figure 1. Funnel plots of the association between age and CCLNM in cN0 PTC.

Supporting Figure 2. Funnel plots of the association between sex and CCLNM in cN0 PTC.

Supporting Figure 3. Funnel plots of the association between size and CCLNM in cN0 PTC.

Supporting Figure 4. Funnel plots of the association between multifocality and CCLNM in cN0 PTC.

Supporting Figure 5. Funnel plots of the association between capsular invasion and CCLNM in cN0 PTC.

Supporting Figure 6. Funnel plots of the association between extrathyroidal extension and CCLNM in cN0 PTC.

Supporting Figure 7. Funnel plots of the association between lymphovascular invasion and CCLNM in cN0 PTC.

Supporting Figure 8. Funnel plots of the association between Hashimoto thyroiditis and CCLNM in cN0 PTC.

Supporting Figure 9. Funnel plots of the association between ipsilateral central lymph node metastasis and CCLNM in cN0 PTC.

## Declaration of interest

The authors declare that there is no conflict of interest that could be perceived as prejudicing the impartiality of the research reported.

## Funding

This work was supported by the Liaoning BaiQianWan Talents Program (No. 2014921033), Natural Science Foundation of Liaoning Province (No. 20180530090), the National Natural Science Foundation of China (No. 81902726), and the Project funded by China Postdoctoral Science Foundation (No. 2018M641739).

## Data availability statement

The data sets used and/or analyzed during the current study are available from the corresponding author on reasonable request.
