# Supplementary figures and images for: Meta-analysis of risk factors for CCLNM in patients with unilateral cN0 PTC
Source: Endocr Connect. 2020 Apr 8;9(5):387–95. doi: 10.1530/EC-20-0058 (PMC7219143; doi:10.1530/EC-20-0058)

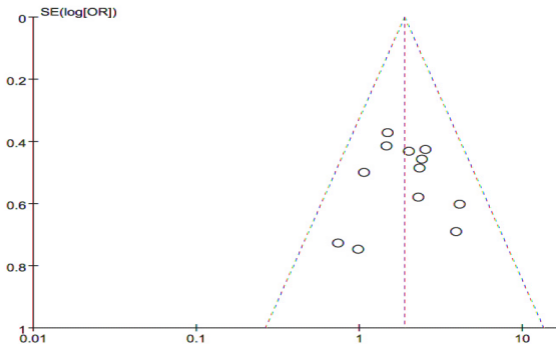

Supplement: Supporting Figure 1. Funnel plots of the association between age and CCLNM in cN0 PTC. [file supplementary_figure_1.pdf]

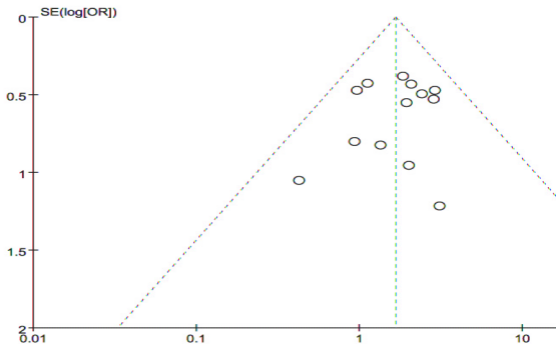

Supplement: Supporting Figure 2. Funnel plots of the association between sex and CCLNM in cN0 PTC. [file supplementary_figure_2.pdf]

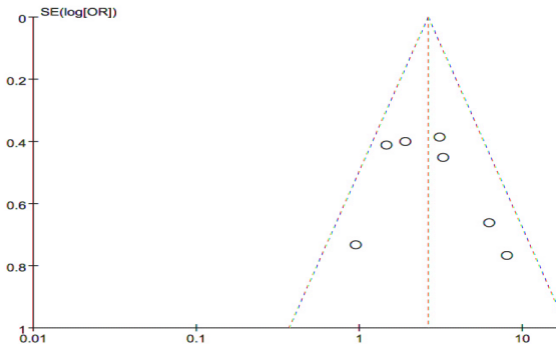

Supplement: Supporting Figure 3. Funnel plots of the association between size and CCLNM in cN0 PTC. [file supplementary_figure_3.pdf]

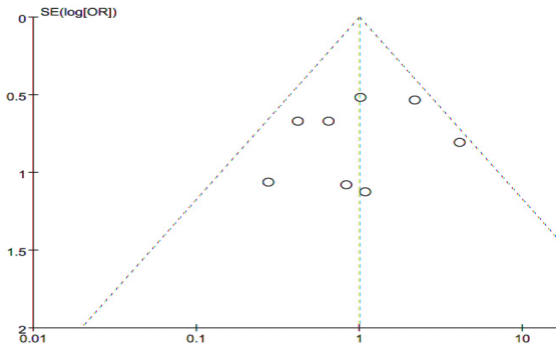

Supplement: Supporting Figure 4. Funnel plots of the association between multifocality and CCLNM in cN0 PTC. [file supplementary_figure_4.pdf]

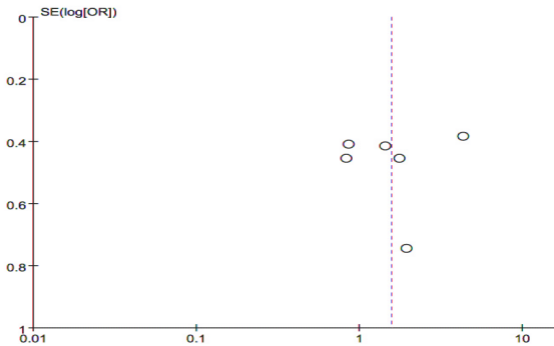

Supplement: Supporting Figure 5. Funnel plots of the association between capsular invasion and CCLNM in cN0 PTC. [file supplementary_figure_5.pdf]

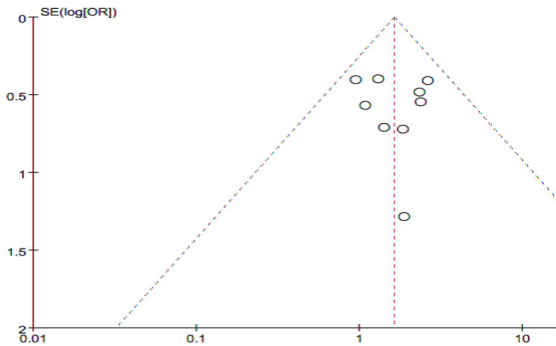

Supplement: Supporting Figure 6. Funnel plots of the association between extrathyroidal extension and CCLNM in cN0 PTC. [file supplementary_figure_6.pdf]

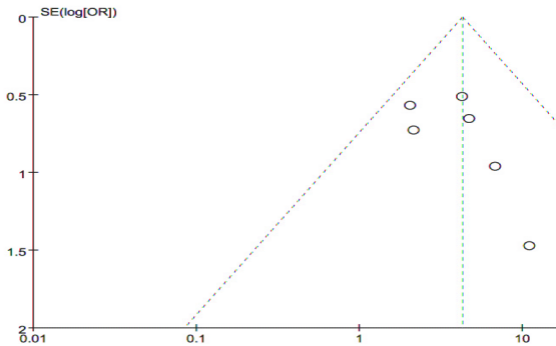

Supplement: Supporting Figure 7. Funnel plots of the association between lymphovascular invasion and CCLNM in cN0 PTC. [file supplementary_figure_7.pdf]

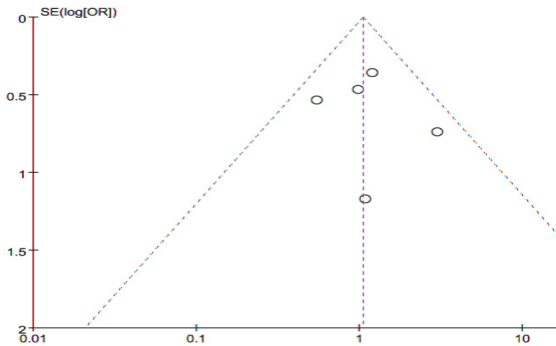

Supplement: Supporting Figure 8. Funnel plots of the association between Hashimoto thyroiditis and CCLNM in cN0 PTC. [file supplementary_figure_8.pdf]

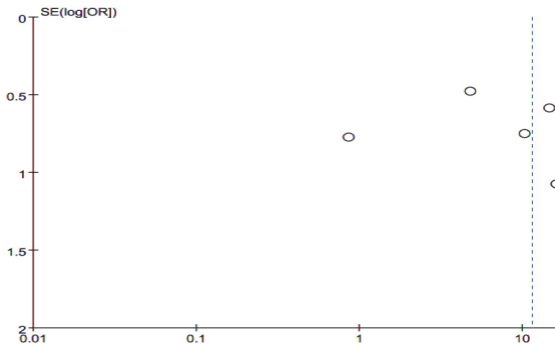

Supplement: Supporting Figure 9. Funnel plots of the association between ipsilateral central lymph node metastasis and CCLNM in cN0 PTC. [file supplementary_figure_9.pdf]
